# Identification of the Major Effector StSROs in Potato: A Potential *StWRKY*-*SRO6* Regulatory Pathway Enhances Plant Tolerance to Cadmium Stress

**DOI:** 10.3390/ijms232214318

**Published:** 2022-11-18

**Authors:** Yeqing He, Guandi He, Fei Lou, Zheng Zhou, Yao Liu, Yule Zhang, Tengbing He

**Affiliations:** 1 College of Agricultural, Guizhou University, Guiyang 550025, China; 2 Institute of New Rural Development, Guizhou University, Guiyang 550025, China

**Keywords:** cadmium tolerance, redox, similar to RCD1 family, regulation pathway, *Solanum tuberosum*

## Abstract

SIMILAR TO RCD-ONE (SRO) family members and transcription factors (TFs) often improve plant antioxidant capacity through interaction and co-regulation and participate in plant resistance to drought and high-salt stress. However, whether SROs are involved in the response to heavy metal stress, especially SRO genes with a specific response and tolerance characteristics to cadmium (Cd) stress, remains unclear. We first identified six SRO genes in the potato genome by PARP and RST domains. Special and conserved StSROs were found, and the spatio temporal tissue-specific expression patterns and co-expression network diagrams of StSROs under the stress of 5 heavy metals were constructed. Second, we identified StSRO6 as a major effector gene (StSRO6-MEG) and StSRO5 as a secondary effector gene (StSRO5-SEG) through a comprehensive analysis. Interestingly, they may hold true for various physiological or stress responses in plants. In addition, using systematic genomics and comparative omics techniques, the key gene StSRO6 that affects the difference in Cd accumulation was discovered, cloned in the low-Cd accumulation “Yunshu 505”, and transformed into the yeast mutant *ycf1* for overexpression. The results proved that StSRO6 could confer Cd tolerance. Finally, through transient expression and in vitro culture tests, we hypothesized that StSROs 5/6 are regulated by the transcription factor StWRKY6 and mediates the reactive oxygen species (ROS) system to confer Cd tolerance. These findings offer a new perspective for understanding the mechanisms underlying Cd tolerance in plants, and simultaneously provide clues for the development of biological agents for preventing and controlling Cd migration and transformation.

## 1. Introduction

Radical-induce cell death1 (RCD1) is a major effector gene (MEG) of abiotic stress in the SIMILAR TO RCD-ONE (SRO) gene family, which interacts with transcription factors (TFs) to regulate plant redox levels in plants and enhance the resistance of plants to abiotic stresses such as drought [1]. SRO gene family members play an important role in regulating plant stress tolerance, in spite of this, research on heavy metal stress have been still pretty scarce. Nevertheless, whether SROs also enhance plant tolerance to heavy metal stress and whether a MEG is involved remain to be explored.

RCD1 is named after clone eight-one (CEO1) in *Arabidopsis thaliana* (*A. thaliana*). CEO1 could restore the antioxidant capacity of mutant yeast strains [2]. Subsequent studies have found that *ceo1* mutants cause rapid cell death in *A. thaliana*; hence, it was renamed radical-induce cell death1 (RCD1) [3]. As the research intensified, RCD1 and its homolog SROs were classified into the plant SRO family. Pinja Jaspers et al. identified AtRCD1 and its 5 homologs AtSROs 1–5 in *A. thaliana* [4]. They found that AtRCD1 is the MEG in the SRO family, manifested as the mutant line *sro1* (mutated AtSRO1 gene, which is the orthologous gene with the highest similarity to MEG-AtRCD1), showed no effect on plant growth and development, and increased the tolerance of *Arabidopsis* to osmosis and oxidation stress [5]. Interestingly, in the background of the *Arabidopsis rcd1* mutant line (MEG-AtRCD1 mutation), a single mutation of AtSROs 1–5 results in severe developmental defects in plants, suggesting the functional compensatory action of MEG and vice effector gene (VEG) between AtRCD1 and AtSROs [6]. However, the phylogenetic relationship between RCD1 and SROs in the SRO family of Solanaceae has not been characterized, and the MEG of SRO family members in different plants that help the plants to cope with heavy metal stress remains undetermined.

Previous studies have found that RCD1 (MEG) and SRO (the RCD1 homolog) proteins in the plant SRO family mostly contain the poly(ADP-Ribose) polymerase (PARP, PF00644) catalytic domain and the C-terminal RCD1-SRO-TAF4 domain (RST, PF12174) [7]. A few SROs, such as RCD1, also contain a WWE domain (PF02825) named based on the position that it is located after three conserved residues W and E (tryptophan and glutamate, respectively) at the N-terminal [8]. Pinja Jasper et al. divided SROs in *A. thaliana* into type A and type B. Among them, type A includes AtRCD1 and AtSRO1, which contain WWE, PARP, and RST domains, whereas other members belong to type B, lacking the N-terminal WWE domain [4]. By analyzing the origin of these three domains, they found that the PARP–RST domain combination is unique to plants, whereas the WWE–PARP domain combination is highly conserved and exists widely in animals, microorganisms, and plants [9,10]. In particular, MEG-RCD1 in plants typically includes a more comprehensive combination of domains, including three domains, especially the WWE domain.

With the discovery of a transcription initiation complex of AtRCD1 and TF, AtRCD1 is suggested to function as a transcriptional cofactor (similar to a TF) by forming a protein complex [11]. Meanwhile, WWE and RST domains are special functional regions that mediate protein interactions. WWE sites may mediate glycosylation or ubiquitination and play a crucial role in signal transduction, DNA repair, and protein stability [12]. RST is the TF interaction site that can mediate the direct interaction between SROs and multiple TF families [13], and thus co-regulate the transcription of downstream genes [14,15]. In addition to that in *Arabidopsis*, the function of SROs in abiotic stress has been reported in rice, wheat, and maize. OsSRO1 transcription in rice is regulated by the TF SNAC1, which enhances the plant antioxidant capacity [16]. Jiang et al. found that TaSRO1 improves the salt tolerance of wheat seedlings by ensuring the stability of the redox system and genome [17]. In maize, ZmSRO1 and TF competitively bind key subunits to form transcription-activated complexes, thereby enhancing maize resistance to abiotic stress, but inhibit anthocyanin synthesis [18].

In our previous studies, we found that cadmium (Cd) accumulation and distribution of the low-Cd accumulation genotype potato variety “Yunshu 505” are regulated by various transmembrane transporters [19]. However, the potential upstream regulatory pathway remains unclear. Transcriptomics subsequently revealed that the TF StWRKY6 (AtWRKY6-like, a key responsive factor in potato Cd stress) and StSROs exhibited co-expression characteristics, especially the SRO family members lack considerable functional redundancy and have a major effector role in maintaining intracellular homeostasis. Therefore, further research on the potential mechanism of potato StSROs and StWRKY6 holds significance and application potential. Based on the foregoing discussion, this study used potato “Yunshu 505“ to explore StSRO characteristics by investigating systemic-comparative omics; toxicology and molecular biology; phylogeny, structure, response model, and function; key stress reaction MEG-StSROs; and potential regulatory pathways of oxidative stress under Cd stress. The aim was to further excavate upstream key genes that may be actively involved in Cd stress, offer a new perspective for understanding the mechanism underlying Cd tolerance in plants, and provide clues for developing biological agents that can prevent and control Cd migration and transformation.

## 2. Results

### 2.1. Genome-Wide Identification and Physicochemical Properties of SROs in Potato

**Figure 1:**
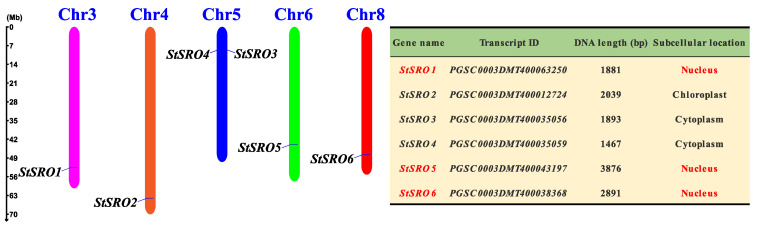
Chromosome location and basic information analysis of StSRO family members. Different colors represent different chromosomes. Chromosome numbers are shown at the top; StSROs were labeled on the left and right sides of the chromosome. The scale on the left shows chromosome length (in Mbp). The table on the right of chromosome shows StSRO family information and subcellular localization analysis.

Through gene annotation files, six StSROs were screened from potato genome database and named StSROs 1–6 according to their positions on chromosomes. The six genes were mainly distributed on five chromosomes and located in the gene dense region. Among them, Chr5 was distributed with two genes, and the other chromosomes contained only one gene. The positions of StSRO3 and StSRO4 were very close, but no tandem duplication existed between the two genes (Figure 1). Analysis of the physicochemical properties of StSRO proteins revealed that the gene lengths ranged between 1467 and 3876 bp (StSRO4–StSRO5), molecular weights ranged between 33.72 and 67.47 KDa (StSRO4–StSRO5), isoelectric points ranged between 5.97 and 9.16 pI (StSRO2–StSRO4), and grand averages of hydropathicity were between −0.454 and −0.218 (StSRO1–StSRO4), indicating that all of them were hydrophilic proteins. Furthermore, the subcellular localization of proteins revealed that only StSROs 2/5/6 of the StSRO family were located in the nucleus, suggesting that StSROs 2/5/6 could be used as a transcription cofactor for regulating downstream gene expression. The specific theoretical properties of StSROs are described in Supplementary Table S3. 

### 2.2. Evolutionary Exploration of SROs in Potato and Different Species

**Figure 2:**
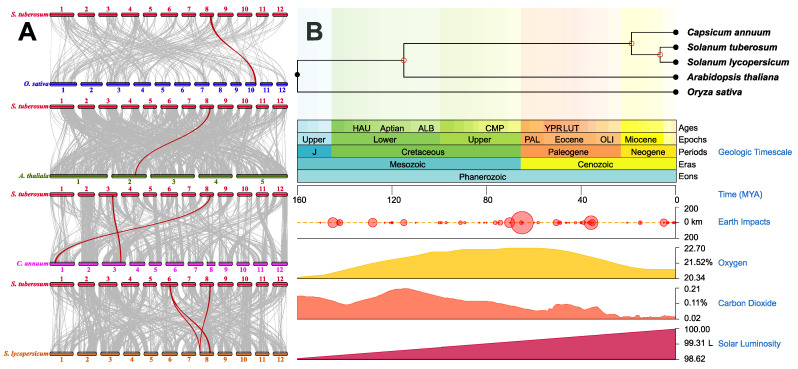
Collinearity analysis of SROs in *S. lycopersicum*, *C. annuum*, *A. thaliana,* and *O. sativa*. (**A**) Different species are represented by chromosomes of different colors. The red line represents homologous pairs in StSROs, and the gray line represents collinear blocks in plant genome. (**B**) Evolutionary trees of *S. tuberosum* and the four species mentioned above. Different background colors indicate when the species diverged. The species evolutionary tree was constructed using the online Timetree software (http://www.timetree.org accessed on 13 November 2022) [21].

The nightshade family is approximately 52 million years old, whereas the *Solanum Tuberosum* (*S. tuberosum*) split approximately 5000 years ago. The potato has a complex evolutionary history, and the origins of its ancestors were influenced by many factors [20]. To further infer the evolutionary origin of the StSRO family and the collinearity among other species, we performed collinearity analysis on *Solanum lycopersicum* (*S. lycopersicum*), *Capsicum Annuum* (*C. annuum*), *A. thaliana,* and *Oryza sativa* (*O. sativa*). In total, seven collinear gene pairs were found in four species. Among them, the most collinear gene pairs (three pairs) were found in *S. tuberosum* and *S. lycopersicum* (Figure 2A), both belonging to Solanaceae, which indicated their high degree of genetic similarity. One particular gene (StSRO6) has continuous collinear gene pairs in *S. tuberosum*, *S. lycopersicum*, *C. annuum*, *A. thaliana,* and *O. sativa*. This may be related to the phylogenetic relationships between the four species (Figure 2B). Its peculiarity indicates that the evolutionary function was conserved, not lost or changed during replication, and seems to preserve ancestral functions.

### 2.3. Phylogenetic Tree Analysis and Classification of SROs in Many Types of Plants

**Figure 3:**
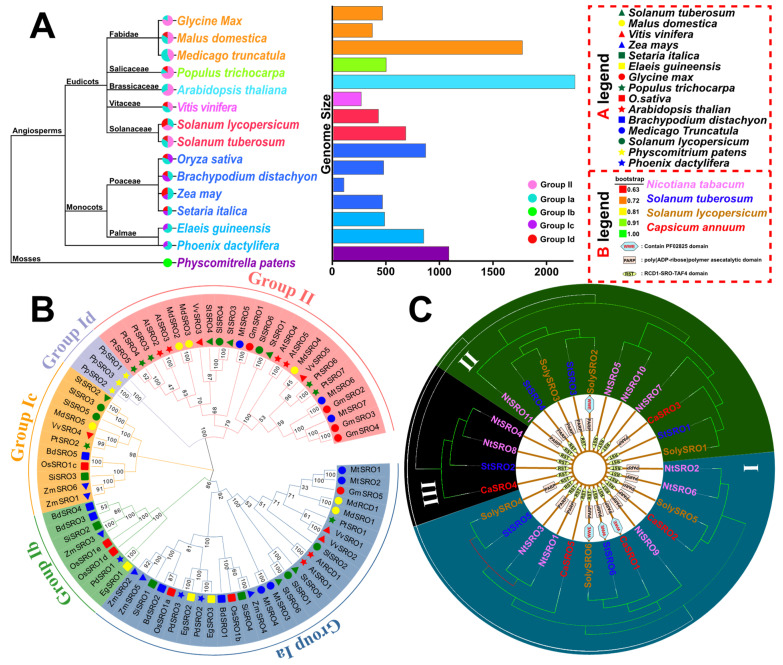
Evolutionary analysis of SRO families in different species. (**A**) The evolutionary relationship and number of SRO families in 15 species, *S. tuberosum* (6), *Malus domestica* (*M. domestica*, 6), *Vitis vinifera* (*V. vinifera*, 5), *Zea mays* (*Z. mays,* 6), *Setaria italic* (*S. italic*, 4), *Elaeis guineensis* (*E. guineensis*, 3), *Glycine max* (*G. max*, 5), *Populus trichocarpa* (*P. trichocarpa*, 7), *O. sativa* (*O. sativa*, 5), *A. thaliana* (6), *Brachypodium distachyon* (*B. distachyon*, 5), *Medicago Truncatula* (*M. truncatula*, 7), *S. lycopersicum* (6), *Physcomitrium patens* (*P. patens*, 3), and *Phoenix dactylifera* (*P. dactylifera*, 3), the color of species represents their taxonomic characteristics, and the size and color of sectors represent the number of SRO families in species and the groups to which they belong. (**B**) Using Neighbor Join (NJ) method in MEGA-X to construct phylogenetic tree, SROs of different species are divided into two groups (Group I and Group II), with different shapes of logos representing different species, as detailed in Supplementary Table S1. (**C**) Phylogenetic tree of the SRO families of Solanaceae, SROs of different Solanaceae species are represented by different font colors, the evolutionary tree shows the conserved domains of corresponding SROs, among which the WWE domain is green, the RST domain is yellow, and the PARP domain is pink, as detailed in Supplementary Table S5.

To better understand the evolutionary history of SROs, MEGA-X software was used for conducting multi-sequence comparison of 77 SRO protein sequences of 15 species, namely eight eudicotyledons, six monocotyledons, and one bryophyte (Figure 3A). Phylogenetic tree analysis showed that the number of SROs increased in different plants, from algae to flowering plants, as the complexity of organisms increased [22]. Most plant genomes contain 5–7 SRO members, and *Elaeis guineensis*, *Physcomitrium patens*, and *Phoenix dactylifera* contain three SRO members. Bootstrap software (1000 repetitions) was used to generate a multispecies phylogenetic evolutionary tree (Figure 3B). The 77 SRO members of 15 species were grouped into group I and group II. Group I contained true dicotyledons and monocotyledons, whereas group II contained only true dicotyledons. Group I was further divided into four subgroups (Ia, Ib, Ic, and Id) according to the branch value (>75) [7]. Among them, AtRCD1/AtSRO1 of *Arabidopsis* and StSRO5/6 of potato were clustered in the same subgroup (group Ia) with close genetic relationship and formed group Ia with SRO homologs of all selected species except *P. patens*. The three PpSROs of bryophytes formed an independent group Id. Only monocotyledons participated in group Ib. In particular, SRO members were widely distributed in potato, with StSRO1, StSRO3, and StSRO4 aggregating in group II, StSRO2 aggregating in group Ic, and StSRO5 and StSRO6 aggregating in group Ia. This indicated that the expansions of the SRO family in potato were relatively conservative. Interestingly, in the phylogenetic tree, only SlSRO2 formed a single branch and had a distant relationship with potato, whereas the SlSRO members that did not form a single branch were most closely related to StSROs.

To further analyze the lineage-specific expansion of the SROs families, we constructed a phylogenetic tree of SRO family members of Solanaceae (potato, pepper, tobacco, and tomato) (Figure 3C). According to the difference in CDS length and domain, all SRO genes were categorized into three types. Class I protein genes were the longest, except for StSRO5 and CaSRO5, which only contained the RST domain. The remaining proteins had PARP and RST domains; StSRO6, SolySRO6, and CaSRO1 also had a WWE domain. Class II proteins were the shortest, and some proteins contained only an RST domain. In Class III, NtaSRO4, NtaSRO8, and StSRO2 contained an RST domain at the N-terminus. The StSRO family members were highly conserved, located in adjacent branches of different subfamilies, and exhibited good genetic relationships.

### 2.4. Gene Structures and Conserved Motif Analysis

Exon–intron structure is the fragment retained by the gene after mRNA shearing, and most exons are coding sequences [23]. In this study, a comprehensive analysis of conserved motifs and phylogenetic trees of gene structures revealed that members of similar gene families clustered preferentially (Figure S1A). According to the structural analysis of the plant SRO families (Figure S1B), most group II members (18/22: 81.82%) had 2–4 exons, and the number of introns was similar to that of exons. Most group Ia members (11/19: 57.90%) had 3–4 exons. Except for OsSRO4, which had four exons in group Ib, other genes only contained two exons. Most group Ic members (5/7: 71.42%) had 1–2 exons. Most SROs contained five introns, among which ZmSRO6 and SiSRO6 contained only one intron. Furthermore, genes on the same branch of the evolutionary tree were structurally similar and their CDS had a similar number of introns. Most group Ia genes contained multiple CDS and introns, indicating that their functions may be highly similar. Interestingly, among the StSRO family, StSRO6 (group Ia) contained four exons, whereas other StSROs contained only two exons, and StSRO6 gene contained the most introns. These results indicate that StSRO6 has more functional diversity than other StSRO family members.

According to the analysis results of exon length distribution (Table S8), in 6 StSRO genes, the number of exons was 3–8. As shown in Figure S1E, the lengths of exons E2 and E3 appeared to be more limited, whereas exon E6 was more variable (1228–2846 bp). The presence of exons E3, E7, and E8 is unique to certain genes. Despite this difference, the lengths of E4 and E5 exons were very similar (E4: 929 bp; E5: 912 bp). We found a common pattern of diversity in the StSRO family using the 3D scatter plot plotted by SigmaPlot (Figure S1F) [24]. The existing exons of the StSRO family have the same length and similar distribution as some exons of *S. lycopersicum*, *C. annuum*, and *A. thaliana* species. In total, 15 motifs were predicted and visualized by using the online site MEME (Figure S1C). SROs of the same subfamily exhibited a similar pattern of conservative motif distribution. The conserved motifs of different subfamilies of SROs differed considerably in composition. The presence of motifs 1/2/4/5 was detected in all SROs. StSRO 5/6 motifs exhibited the same distribution and quantity, they are also the most motif proteins (1/2/3/4/5/6/7/8/9//11/12/13).

The NCBI-CDD tool was used to analyze the structure of SRO proteins [25]. Almost all SROs exhibited RST and PARP domains (Figure S1D), among which the RST (RCD-SRO-TAF4) domain was mainly located in the C-terminal tail. The TAF4 (TATA box binding protein TBP coupling factor 4) is a component of several polymeric protein complexes including the transcription factor complex TFIID [26]. The PARP domain functions to repair DNA damage following environmental stress [27]. These two structural domains are very important to the basic function of SROs. In particular, AtRCD1/SRO1 and StSRO6 in group Ia contained complete WWE-PARP-RST domains, indicating that StSRO6 and AtRCD1/SRO1 may have similar functions and both play a major role in heavy metal-induced oxidative stress.

### 2.5. Expression Profile of SROs in Different Potato Tissues and Expression Response Analysis of Five Heavy Metals under Stress

To study the enrichment and expression of StSROs in different tissues, transcriptome data were downloaded and visualized (Figure S2). According to the results, StSROs were mainly divided into two types. The first type was expressed specifically in some tissues, and the second type was expressed in all 15 tissues. Members of the first type accounted for more than 33%, and the second type, that is, StSROs 1/4/5/6 may be redundant genes; they may also be acting as the signal transduction function gene of stress. StSRO2 expression was relatively low in each tissue, whereas the StSRO6 transcription level was relatively high in all tissues, with the TPM value as high as 148 in lumpy roots. The StSRO family exhibited low transcription levels in the prostrate and tuber and moderate and low transcription levels in flowers. In addition, StSRO1 and StSRO6 exhibited relatively high transcription levels in flowers and pistil and may be involved in potato growth and development.

**Figure 4:**
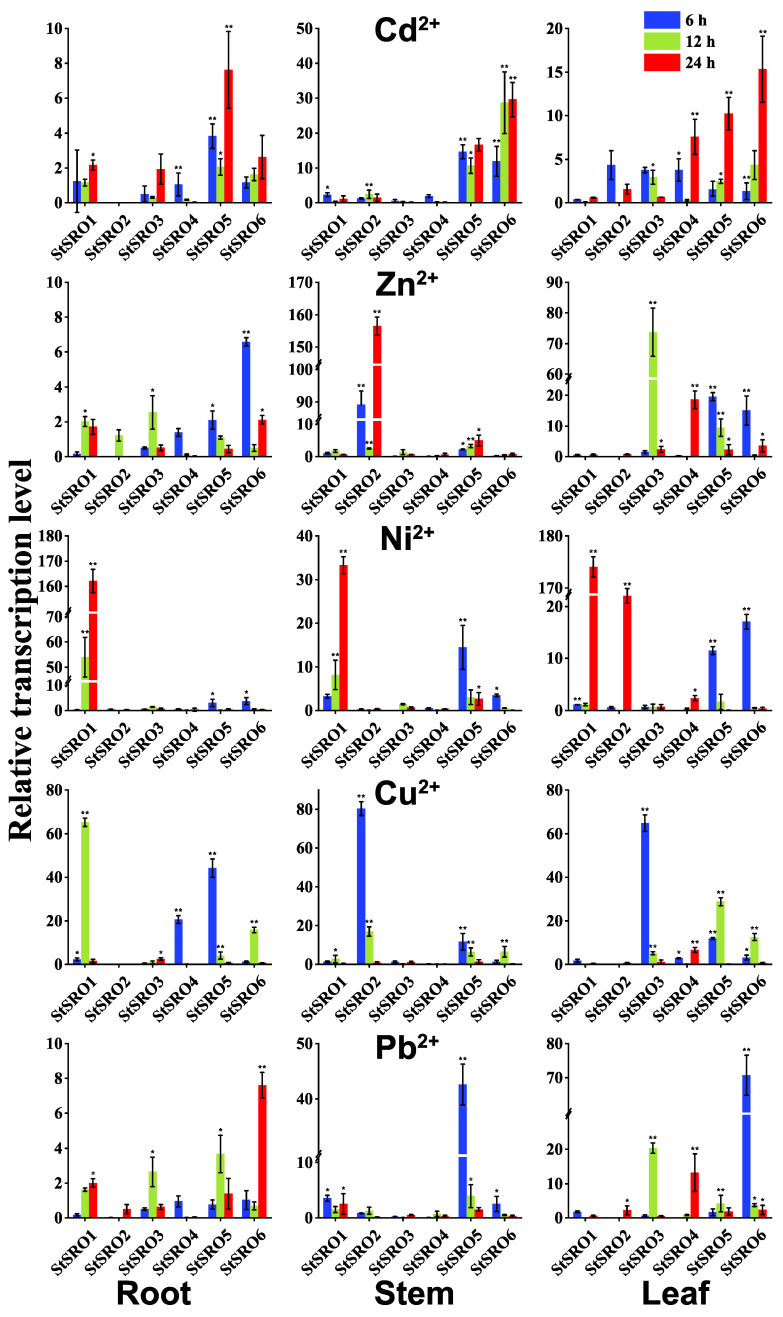
Expression levels of StSROs in roots, stems, and leaves under different periods of heavy metal stress (6/12/24 h), the final data were the average of three biological replicates (±SD). The vertical lines on the bar chart indicate the standard deviation, as detailed in Supplementary Table S9 (* *p* < 0.05 significant difference at the 0.05 level, ** *p* < 0.01 difference extremely significant at the 0.01 level).

After exposure to five heavy metals, the overall expression enrichment of six genes in the StSRO family was studied. As shown in Figure 4, at least one StSRO family member responded positively to the treatment of the five heavy metals. StSROs exhibited different expression patterns at different time points, and the upregulation trend of most genes with time was as follows: 24 h > 12 h > 6 h. The expression of StSROs in different tissues showed the following trend: root > leaf > stem. Some genes were upregulated under stress of some heavy metals. For example, under Cd stress, StSRO6 expression was upregulated approximately 29-fold in stems and 15-fold in leaves. Under Zn stress, StSRO2 expression was upregulated approximately 156-fold in leaves. Under Ni stress, StSRO1 expression in the roots, stems, and leaves was upregulated by approximately 162-, 33-, and 17-fold. Under Cu stress, *StSRO3* expression in leaves was upregulated approximately 64-fold. Under Pb stress, StSRO6 expression was upregulated approximately 202-fold in leaves. Interestingly, the StSRO5 expression level in potato RNA-Seq was low but significantly increased after Cd stress, suggesting that Cd can activate StSRO5 expression. Notably, StSRO6 responded positively and was highly upregulated under stress of more than three heavy metals at any time point, especially in leaves exposed to five heavy metals. StSRO6 expression was highly induced.

### 2.6. StSRO Co-Expression Framework

**Figure 5:**
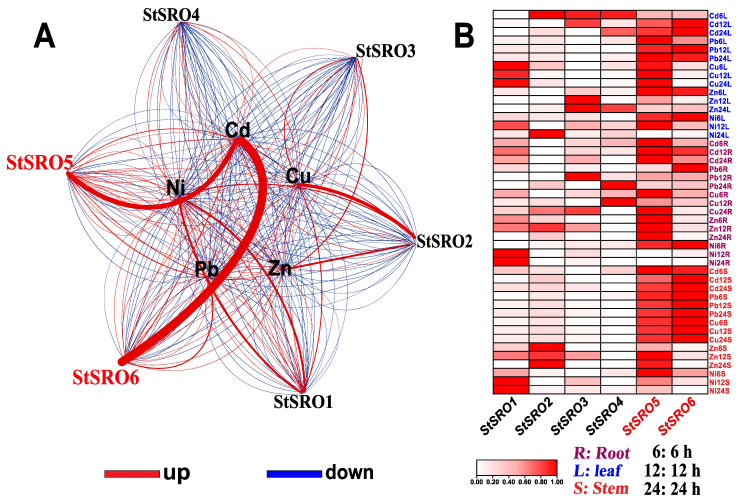
Co-expression network analysis of StSRO family. (**A**) Co-expression network analysis of StSROs under stress of five heavy metals. The ability of gene expression levels is shown in red (strong) and white (weak), respectively. (**B**) Expression patterns of StSROs in different tissues, changes in gene expression levels are shown in red (high) and white (low). Root, stem, and leaf are abbreviated to R, S, and T, respectively, and time (h) is denoted by 6, 12, and 24, respectively, as detailed in Supplementary Table S10.

The co-expression network is a type of analysis method that uses gene expression data to construct the correlation network between genes and thus excavate gene function [28]. Therefore, Cytoscape software was used to construct a co-expression network based on the differential expression levels of StSROs under the stress of five heavy metals (Figure 5). StSROs are significantly induced or inhibited by various heavy metals; Cd and Pb treatment significantly induced StSRO6 expression. Ni and Pb treatments significantly induced StSRO1 expression. Conversely, the same heavy metal can simultaneously induce the expression of multiple StSROs. For example, Cd treatment induced two genes (StSRO5, StSRO6). In addition, a more detailed co-expression pattern of StSROs was observed (Table S9). Under Cd and Pb stress, StSROs 5/6 were positively upregulated in leaves. StSRO6, which is the representative gene and highly responsive to Cd stress in different tissues, was selected for further studies.

### 2.7. Prediction of Protein Folding Structure

Proteins, as the basis of living matter, rarely act individually but interact in a very well-defined manner to coordinate almost all cellular processes [29]. Therefore, to understand the structure and ion transport function of StSRO proteins, we used Alphafold2 released by the Deepmind team to predict the StSRO protein structure (Figure S3). The three-dimensional structures of proteins in the same subfamily were very similar. In addition, to better comprehend the inner workings of cells, we predicted StSRO protein–protein interaction models. As shown in Figure S3B, the possibilities of interaction between StSRO5 and StSRO6 and StSRO2 and StSRO3 were far higher than that between other combinations, whereas the possibility of interaction between StSRO1 and StSRO3 was low. This may be because they act alone or require other protein molecules to coordinate with each other to achieve complex cellular functions. This result provided research direction for the verification of the interaction between proteins in the next step.

### 2.8. StSRO Regulatory Network Analysis

**Figure 6:**
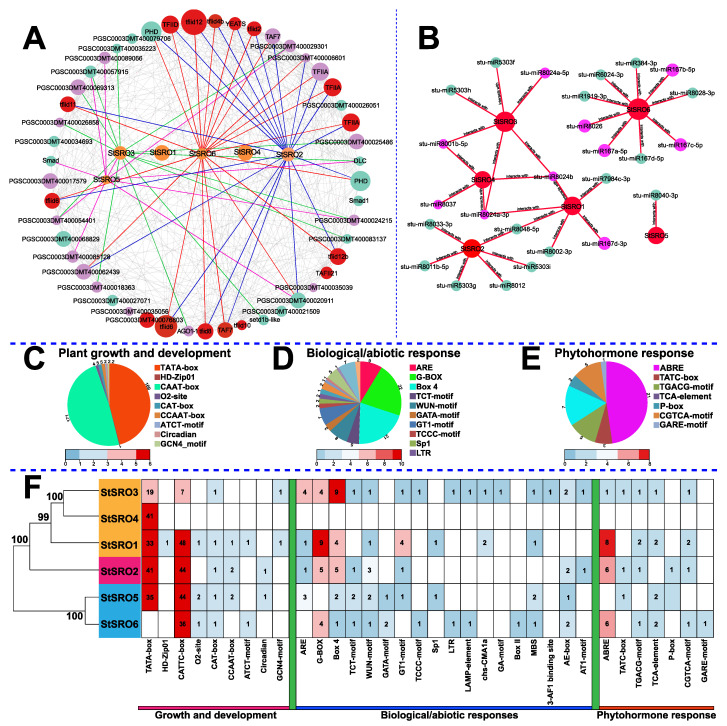
Interaction network diagram of StSROs and motif analysis and prediction of cis-acting elements of promoter. (**A**) Network analysis of interactions between StSROs and other proteins. Each circle represents a protein, the size of the circle represents the number of interactions between StSROs and other proteins, the connection between the two circles represents the interaction between the two; the protein of StSROs were represented by orange circles, and related proteins are represented by green circles. Transcription factors are indicated by red circles and unlabeled proteins are indicated by purple circles, as detailed in Supplementary Table S10. (**B**) Targeted interactions between StSROs and miRNAs. StSRO family members were represented by red circles, and the number of connections between each circle indicates the number of interactions. Abiotic stress-associated miRNAs were represented by purplish red circles, and biological stress-associated miRNAs are represented by green circles. (**C**) Proportion of plant growth and development. (**D**) Proportion of plant hormone responsive cis-elements. (**E**) Proportion of the number of cis-elements associated with abiotic stress. (**F**) Cis-acting elements of StSROs, as detailed in Supplementary Table S12.

To further understand the functions of SROs in potatoes, we predicted the interactions between StSRO proteins by using the STRING online database and drew the interaction network diagram (Figure 6A). According to the results, StSROs 1/4 did not interact with other proteins, and StSROs 2/3/5/6 synergistically regulated similar physiological functions with other proteins. A total of 64 wires were present; an interaction was observed between StSRO3 and StSRO5. Of these, StSROs 2/3 interacted with 18 proteins, StSRO6 interacted with 14 proteins, and StSRO5 interacted with only 10 proteins. Proteins that interacted with the StSRO family can be classified into three types: genetic factors, transcription factors, and noncharacteristic proteins. The epigenetic factor setdeb1 mainly interacts with StSRO3. StSROs also interact with a large family of transcription factor proteins, including eukaryotic transcription factors TFIID4B and TFIID10, to promote RNA polymerase binding to promoters. Moreover, some unlabeled proteins in the protein interaction network diagram exert obvious synergistic effects with StSROs proteins, although their functions remain unclear.

The diversity and evolutionary conservation of microRNAs (miRNAs) determine the importance and universality of physiological and biochemical functions [30]. Therefore, we predicted the targeted regulatory relationship between miRNAs and StSROs through the psRNATarget database (Figure 6B, Table S11), providing information for the regulatory mechanism of StSROs. By regulating the network connection distribution, all StSRO family members were predicted to have a targeted regulatory relationship with miRNAs. StSRO6 was the most targeted SRO gene, which was predicted to be regulated by nine miRNAs and has the strongest regulatory effect. StSRO5 was only regulated by Stu-miRNA8040-3p. Most miRNAs target at a single StSRO, and only Stu-miRNA8048-5P regulates StSROs 1/2. Stu-miRNA8001-5p regulates StSROs 3/4. In particular, StSROs 1/3/4 can be simultaneously regulated by both Stu-miRNA8024b and Stu-miRNA8024A-3p. Some conserved miRNAs (Stu-miRNA8048-5P, Stu-miRNA8024b and Stu-miRNA8048-5P) have crucial roles in plant growth and development, resistance to abiotic stress, and genome integrity, and they participate in some physiological processes. In summary, the results of the aforementioned protein interaction network and microRNA-targeted regulation provide more research targets on the StSRO function.

Cis-acting elements are short DNA sequences with transcriptional regulation function that can affect gene expression activity [31]. To study the response of StSROs to various signal factors, various cis-acting elements were searched with 2000-bp sequences upstream of their transcription start sites (Figure 6F). Many hormone response elements (ABRE, TATC-BOX, P-box, etc., 7), growth- and development-related elements (MBST, CCAAT-BOX, CAT-box, etc., 9), and biological/abiotic stress response elements (ARE, G-BOX, Box4, etc., 18) were found. The number of cis-acting elements in the StSRO family varies greatly. StSRO3 has the most types of cis-acting elements (24 types), but StSRO2 has even higher and the maximum number (118 types) of cis-acting elements. StSROs 5/6 shared numerous CATTC-box cis-acting elements and was the transcription initiation site. In particular, StSRO4 only contains elements related to the growth and development of TATC-BOX. In conclusion, most StSRO family members contain these three types of cis-acting elements, suggesting the potential role of StSRO family members in potato growth and development, as well as in multiple hormones and stresses.

### 2.9. Effect of Agrobacterium-Mediated Transient Expression of StWRKY6 on Transcription of StSROs 5/6

StSROs 5/6, as an MEG and a VEG in the StSRO family, plays an important role in Cd stress. According to our previous study, StWRKY6, a member of the StWRKY family, is closely related to Cd stress. To verify whether StSROs have Cd tolerance under the regulation of transcription factors, transient transfection experiment was performed. First, the Agrobacterium-infected strain was prepared (Figure S4A). The leaf phenotypes of potato soil culture seedlings and tissue culture seedlings were observed before and after infection (Figure S4B,C). The expression of StSROs 5/6 and StWRKY6 genes after Agrobacterium infection was determined through qRT-PCR (Figure S4D,E). The results showed that under Cd^2+^ stress, the expression of StSROs 5/6 was positively correlated with that of StWRKY6 in potato soil culture seedlings and tissue culture seedlings. The expression was highest at 24 h and then showed a downward trend. In particular, the expression level of the tissue cultured plantlet was slightly higher than that of the soil cultured plantlet. This might be because the tissue cultured plantlet was not affected by external bacteria and viruses. Based on the variation trend of expression levels, we speculated that StWRKY6-StSROs 5/6 may be located in the upstream of the same pathway and regulate the transcription of downstream genes, thus conferring Cd tolerance to potato (Figure S4F).

### 2.10. In Vitro Culture and Determination of Cd Tolerance of Potato Leaves

To further verify the relationship between StSROs 5/6 and StWRKY6, potato leaves before and after transient transfection were placed in Hogland solution with different Cd^2+^ concentrations for in vitro culture [32] (Figure S5A). The growth state of the leaves before and after infection was basically the same on the medium containing 0 μmol/L CdCl_2_. With an increase in Cd^2+^ concentration, the degree of yellowing of the leaves after infection was considerably decreased compared with that before infection. These results indicated that Cd stress aggravated the destruction of the chloroplast ultrastructure and disturbed ion homeostasis, leading to the accumulation of reactive oxygen species (ROS) in leaves (Figure S5C). Therefore, to verify the aforementioned phenomenon of leaf chlorosis, levels of peroxidase (POD) and superoxide dismutase (SOD), which are two key enzymes in the ROS scavenging process and are induced by heavy metals, were measured [33]. POD and SOD activities in leaves increased significantly with an increase in the Cd^2+^ concentration (Figure S4B). At the same time, the dead cells of potato leaves treated with 100 μmol/L Cd for 24 h were observed with the propidium iodide (PI) dye, and red fluorescence represented the index of cell damage. As shown in Figure S5D, the red fluorescence signal observed in the leaves after transient transfection was weak. The result indicated that the degree of Cd damage to the plant leaves before the infestation was greater than that after the infestation. Meanwhile, to explore the effect of Cd accumulation in potato roots after transient transfection, dithizone staining was used to analyze the roots. Cd can be combined with dithizone to form a reddish-brown complex, and the distribution of Cd in potato roots can be observed under a microscope. After 100 μM Cd treatment for 4 days, the transiently transfected potato roots contained more red-brown complexes. Thus, Cd content in the root system increased after infection, indicating that the infected root system had a cumulative effect on Cd content. Based on the aforementioned studies, we preliminarily speculated that potato Cd tolerance may be due to the ROS pathway mediated by StWRKY6 and StSROs 5/6, which effectively removes redundant harmful substances in cells after Cd stress, thus producing a positive tolerance effect.

### 2.11. Verification of Cd Tolerance Function of the Heteroexpressed StSRO6 Gene in Yeast Cells

**Figure 7:**
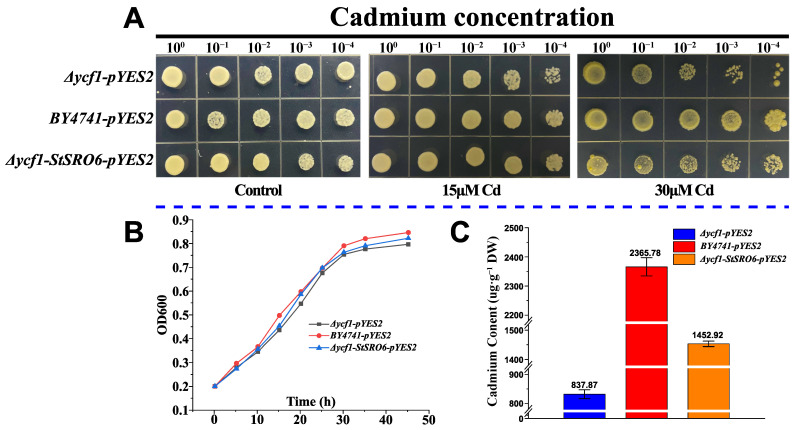
Analysis of Cd tolerance function of StSRO6 heterologous expression in eukaryotic cells. (**A**) Wild-type yeast strain BY4741 (positive control), cadmium-sensitive mutant *Δycf1* (negative control), and overexpressed *StSRO6*-*Δycf1* strain were dilution (10^0^, 10^−1^, 10^−2^, 10^−3^, 10^−4^) cultured in different Cd content (0, 15, and 30 μM) on synthetic galactose-uracil (SG-U)deficiency minimal medium, thus after 7 days, the growth phenotypes of the different strains were observed. (**B**) Biomass changes of three yeast strains at different time points. (**C**) Detection of Cd content in three yeast strains after stable growth (7 d).

After transferring the recombinant plasmid (OEStSRO6-pYES2) into the *Δycf1* mutant strain (Cd-sensitive yeast strain), the Cd tolerance characteristics of different strains from strong to weak were determined (Figure 7A,B). Under 15 μm Cd treatment, the order of Cd tolerance was as follows: BY4741 > OE-StSRO6 > *Δycf1*. Increasing Cd concentration to 30 μm showed the characteristic more significantly. This indicated that after StSRO6 heterologous gene overexpression in the *Δycf1* yeast strain (background interference was eliminated), the translated protein can still programmatically regulate the transcription of some genes, thus enhancing the Cd tolerance of yeast cells. Notably, StSRO6 could not restore Cd tolerance of the *Δycf1* strain to the level of wild-type BY4741, suggesting that other regulatory pathways enhance Cd tolerance in cells. Nevertheless, interestingly, StSRO6 could not only enhance the Cd tolerance ability of strains but also reduce Cd accumulation in strains (Figure 7C). We speculated that the StSRO6 protein is a transcription cofactor that activates or inhibits the expression of related genes (such as ion pump efflux, ROS oxidative stress, and other genes) to achieve the characteristics of high tolerance and low accumulation with Cd stress in eukaryotic cells. 

## 3. Discussion

### 3.1. Special StSROs 5/6 in the StSRO Family

Through a systemic-comparative omics analysis, we found that the VEG of the StSRO family was StSRO5 and the MEG was StSRO6, and their special characteristics are summarized in Figure S6. Notably, StSRO6 is conserved in the analyzed species, namely *S. lycopersicum*, *C. annuum*, *A. thaliana*, and *O. sativa*, all with significantly correlated collinear gene pairs (Figure 2A). Among them, OsRCD1 has been proven to be the MEG. The phylogenetic tree clarified that AtRCD1/AtSRO1 and StSROs 5/6 proteins were located in the same cluster (Figure 3B), with close genetic distance and high affinity. AtRCD1 was MEG, and AtSRO1 was VEG. *A. thaliana* mutant *rcd1* seedlings exhibited sensitivity to Cd stress [33], suggesting that StSROs 5/6 is involved in response to this stress. To verify this finding, we analyzed the transcriptome data of StSROs in 15 potato tissues (without Cd stress treatment, Figure S2) and found that the transcriptional levels of StSRO6 were high in all tissues, whereas those of StSRO5 were low, consistent with the MEG and VEG expression patterns in most species. Interestingly, 100 mg/kg Cd-treated potato can significantly induce the upregulation of StSROs 5/6 expression (Figure 4). We speculated that under the stress of high Cd concentration, MEG-StSRO6 could not clear excessive free radical redundancy in cells, thus activating VEG-StSRO5 to increase the antioxidant capacity of cells. This is a typical function compensation phenomenon of VEG to MEG. However, whether StSROs have similar expression patterns for other 2-valent ions remains unclear. We analyzed the co-expression of 5 heavy metals (Cd, Cu, Zn, Pb, and Ni) (Figure 5) and found StSROs 1–4 have different heavy metal substrate specificity inducement features. Surprisingly, StSROs 5/6 were significantly activated and transcribed under stress of the 5 heavy metals, suggesting that StSROs 5/6 are genes with a key role in heavy metal stress. We then analyzed the structural characteristics of StSROs. In particular, StSRO6, AtRCD1, and OsRCD1 proteins are similar and have a complete WWE-PARP-RST domain [4,17]. This may determine the MEG function of StSRO6, enabling it to interact with TFs and participate in various abiotic stresses and growth and development stages of plants. In conclusion, we speculate that the StSRO6 protein expressed in the nucleus has a major upstream regulatory role in Cd stress.

### 3.2. OE-StWRKY6 Leads to Co-Expression of StSROs 5/6 to Enhance Potato Cd Tolerance

MEG-SROs (e.g., AtRCD1 and OsRCD1), as recognized transcription co-regulators, bind to TFs in various ways to perform their functions. StSRO6 contains the WWE-PARP-RST domains, whereas StSRO5 contains only the RST domain. The RST domain is the interaction site between StSROs 5/6 and TFs, which coordinately regulates the transcription of downstream genes. The special WWE domain drives the modifications of StSRO6 such as glycosylation and ubiquitination to maintain the stable expression of downstream genes. *Arabidopsis* AtRCD1 interacts with the TF AtWRKY70 and protein kinase AtSOS1 and mediates dependent transduction pathways to regulate cell apoptosis and plant salt tolerance, respectively [34,35]. Our transcriptomic data revealed that the TF StWRKY6 and StSROs 5/6 were co-expressed in time series after Cd stress. Therefore, we speculate that TF StWRKY6 and StSROs 5/6 might be located in the upstream of the same pathway to regulate the transcription of downstream genes, thus conferring potato Cd tolerance. Transient transfection experiments of overexpressing StWRKY6 (OE-StWRKY6) (Figure S4) indicated that the StSROs 5/6 transcriptional levels were positively regulated by *StWRKY6*. In vitro culture of potato leaves (Figure S5) revealed that the enhanced transcription of StSROs 5/6 could offset oxidative stress induced by free radicals on leaf cells, thereby reducing the extent of leaf chlorosis. This may be because StWRKY6 and SROs 5/6 jointly mediate the ROS pathway, which effectively removes redundant harmful substances in cells after Cd stress, thus producing a positive tolerance effect. However, the intracellular Cd-tolerant function of MEG-StSRO6 remains to be further verified. Therefore, we then independently heteroexpressed StSRO6 into yeast for cytological experiments to exclude the background error and explore the intracellular Cd tolerance characteristics of the StSRO6 protein.

### 3.3. Potato MEG (StSRO6) Can Restore Cd Tolerance of Δycf1

The Cd-sensitive yeast strain (*Δycf1*), a mutant strain with yeast Cd factor 1 (*YCF1*) gene deletion, exhibited a growth-restricted phenotype under Cd stress. We heterologously expressed MEG-StSRO6 in the *Δycf1* strain (Figure 7) and found that the StSRO6 (MEG) expression could restore *Δycf1* tolerance to Cd and effectively reduce the Cd content in cells. The protein translated in the yeast, where the potato StSRO6 gene was located, can enhance the ability of cells to tolerate Cd poisoning; this also confirms the result that the *Arabidopsis rcd1* mutant is sensitive to Cd stress [33]. MEG-SRO (including yeast RCD1) was among the other conserved proteins in eukaryotes [13,36]; this is the main condition for the heterologous expression of StSRO6 in yeast to continue to perform its function. We hypothesized that the complete domain contained in StSRO6 allows it to act as a new type of transcription or modification cofactor, thereby regulating the complex and fine stress resistance behavior of cells. For example, under Cd stress, controlling the expression of genes encoding the Cd ion efflux pump, chelation, or transport allows the reduction in the content of Cd in cells. Alternatively, ubiquitination or glycosylation modification of redox stress-related kinases, TFs, and other proteins enhances cell tolerance to Cd toxicity. In a word, the molecular mechanism underlying Cd tolerance in plants with StSRO6 has been only preliminarily discovered and needs to be explored further.

### 3.4. A possible New Pathway for Potato to Cope with Cd Stress

**Figure 8:**
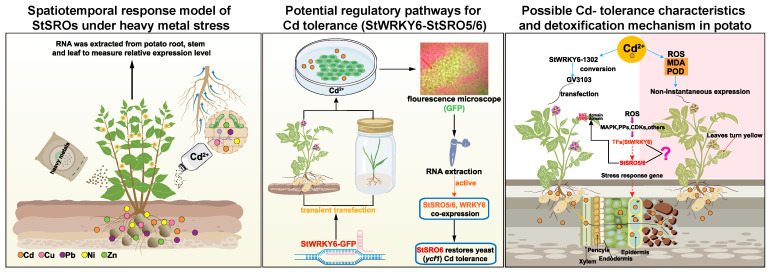
Test design, methods, and conclusions of StSROs identification and functional verification under Cd stress response.

A potential ROS-StWRKY6-SRO5/6-genes regulatory pathway was discovered after Cd stress in potato (Figure 8). As Cd stress activates the ROS system, the release of signals like receptors or kinases increases the StWRKY6 and StSROs 5/6 transcription levels. Furthermore, the two increase their own expression through signal amplification or interaction or jointly regulate the expression of downstream Cd tolerance- and oxidative stress-related genes. The longitudinal and lateral transportation of Cd in plants can be reduced through efflux, chelation, and compartmentalization, thereby improving the scavenging efficiency of intracellular free radicals, and reducing Cd toxicity. We identified potato Cd tolerance genes, such as StABCs [37], StHMAs [38], StOPTs [39], StNRAMPs [40], StMTPs [41], and StMATEs [42], which can transport or sequester Cd^2+^ to control the tissue-specific distribution of Cd^2+^, bio-availability, and vacuolar bearing. Ahlfors et al. found that the *Arabidopsis* rcd1 mutant exhibited abnormal expression of more than 500 genes [43], which means that transcription of the Cd tolerance genes identified in this study may be regulated by the StWRKY6-SRO5/6 module, thus forming a set of the refined management mode and providing a comprehensive defense mechanism for plants. However, for the in-depth study of signal transduction, protein interaction, regulatory network, and other multiaspect mechanisms, scientists from different fields need to work in collaboration. We could only provide the research direction with MEG-StSRO6 as the entry point. Of course, this discovery can be used as a key upstream breakthrough point for studying Cd stress in plants and is expected to make progress in future research.

### 3.5. Conclusions

For the first time, we studied the overall characteristics of the SRO gene family in potato to understand the evolutionary trend, expression profile, and functional speculation of these SRO genes in potato. The study findings will shed light on the role of these genes in plant response to heavy metal stress. Using various biological methods, potato was found to regulate plant tolerance to Cd through the ROS-StWRKY6-SROs 5/6 gene pathway. Date resources of the new target MEG-StSRO6 are a key breakthrough that advance our understanding of the Cd tolerance mechanism in plants, laying a foundation for further exploration of protein interaction and regulatory network under heavy metal stress, plant signal transduction, and candidate proteins for developing biological agents that can prevent Cd migration and transformation.

## 4. Materials and Methods

### 4.1. Toxicological Test and Treatment Method

The potato variety “Yunshu 505” with low Cd accumulation genotype was used as the material. After treatment with 1 mg/kg gibberellin (to break the dormancy of potato tubers and promote germination) for 2 weeks, the buds were 2–4 cm long and transplanted into vermiculite for pot experiment. The perlite ratio was 1:1 and the diameter was 16 cm. Add nutrient solution once every 3 days. The formula of the nutrient solution referred to MS medium, and the plants growing at (24 ± 2) °C were treated with five heavy metal stresses. Cd chloride, copper acetate, nickel nitrate hexahydrate, lead acetate trihydrate, and zinc sulfate heptahydrate were weighed separately. The solution was prepared with a final concentration of 100 mg/kg [40], which was the maximum suitable concentration that the “Yunshu 505” could tolerate, and could activate the expression of most resistance genes, and then the plants under the same growth conditions were subjected to stress test (pure water as control, biological repeat three times). The roots, stems, and leaves of 0 h, 6 h, 12 h, and 24 h heavy metal stress were collected as samples, wrapped and labeled with tin foil, and stored in a refrigerator at −80 °C after liquid nitrogen quick-freezing.

### 4.2. Identification, Basic Characteristics and Interspecies Collinearity of SRO Family Members

Start by downloading *S. tuberosum* proteome file (Ensembl Plants plant genome: http://plants.ensembl.org/info/data/ftp/index.html, accessed on 13 November 2022) [44], download the Hidden Markov Model (HMM) of the SROs domain [WWE domain (PF02825), PARP domain (PF00644) and RST domain (PF12174)] from the Pfam database (http://pfam.xfam.org/, accessed on 13 November 2022) [45], and the use of the HMMER (V3.1) script identification [46]. In addition, to further screen StSRO family members, SMART (http://smart.embl.de/, accessed on 13 November 2022) [47], NCBI-CDD (https://www.ncbi.nlm.nih.gov/cdd/, accessed on 13 November 2022) and PFAM (http://pfam.xfam.org/, accessed on 13 November 2022) data were used to verify the existence of the domain [48]. Six StSRO family members were obtained after removing proteins with low molecular weight and no domain. The location information of StSROs on chromosomes was obtained according to gene annotation files, and the online platform MapGene2Chrom Web v2 (http://mg2c.iask.in/mg2c-v2.0/, accessed on 13 November 2022) was used to draw the location map of StSROs on chromosomes [49]. Collinearity between *S. tuberosum*, *S. lycopersicum*, *C. annuum A. thaliana,* and *O. sativa* SROs were analyzed using MCScanx [50]. The physicochemical properties of StSRO family members were analyzed using ExPASy (http://www.expasy.org, accessed on 13 November 2022) interactive platform, and the online tool WoLF PSORT (https://wolfpsort.hgc.jp, accessed on 13 November 2022) was used for subcellular localization [51].

### 4.3. Interspecies Phylogenetic and Evolutionary Analysis of SROs

In order to analyze the evolution and function of SROs among 15 species, the sequence files of the corresponding species of SROs were downloaded according to the database, and the ClustalW function in MEGA-X was used to compare with the StSROs proteins respectively. Then, the phylogenetic tree was constructed using the Neighbor-Joining (NJ) method in MEGA-X software, and 1000 bootstrap replicates were set up [52]. The constructed evolutionary tree was imported into the online software Evolview v3 (http://www.omicscalass.com/article/671, accessed on 13 November 2022) for visualization [53]. Patterns and functionally annotated SROs in Solanaceae were matched to adjacent branches of StSROs for functional analysis.

### 4.4. The Functional Analysis of SRO Family Members was Carried out Based on Structures, Motifs, and Cis-Acting Elements

In this study, based on SRO GFF3 annotation files of 14 different species, intron–exon and UTR localization information of SROs were obtained using Bio-Linux system, and the analysis results were imported into Tbtools for visualization [54]. Conservative motifs were analyzed using the default parameters of MEME Suite (http://meme-suite.org/tools/meme, accessed on 13 November 2022) [55]. The 2000 bp upstream promoter sequence region of each member of the StSRO family was extracted from the potato genome (http://plants.ensembl.org/Solanum_tuberosum/Info/Index, accessed on 13 November 2022) using Tbtools software, which was used to predict the cis-acting elements of the StSROs.

### 4.5. StSROs under/without Heavy Metal Stress and Co-Expression Network Analysis of Five Heavy Metals

For analysis without Cd stress (normal growth), transcript levels of StSROs in 15 plant tissues at growth and developmental stages (data sources, https://www.ebi.ac.uk/arrayexpress, accessed on 13 November 2022) were analyzed [56]. Extract the RNA of the sample obtained in 2.1 and construct a cDNA library, and detect the effects of five heavy metals (Cd, Pb, Zn, Ni, Cu) on the expression patterns of StSROs 1–6 by using the specific real-time fluorescent primers of StSROs CDS (Supplementary Table S2, synthesized by Shanghai Sangong Bioengineering Co., Ltd., Shanghai, China). The internal reference gene was actin, the final data were the average of three biological replicates (±SD). The vertical lines on the bar chart indicate the standard deviation, SPSS 22.0 were used to analyze and process the data. One-way analysis of variance (ANOVA) and LSD were used for multiple comparisons of significant differences (*p* < 0.05) and (*p* < 0.01). The kits used included total RNA extraction reagent (Noviz Biotechnology Co., Ltd., Nanjing, China) and StarScript II degenomic DNA reverse transcription master mix (Kang Runcheng biological technology Co., Ltd., Beijing, China.). The relative expression levels of StSROs were calculated by 2^−ΔΔCt^ method. Finally, Origin 2019B 64-bit, Tbtools and Cytoscape were used to construct the co-expression network map of each gene [57]. 

### 4.6. StSRO Family Interaction Network Analysis and Proteins Tertiary Structure Prediction

Analysis of StSRO Family Interaction Network in order to study the possibility of StSROs proteins interacting with other proteins, StSRO family protein sequences were uploaded to the Search Tool for the Retrieval of Interacting Genes/Proteins (STRING) database (https://string-db.org/, accessed on 13 November 2022) [58]. Potato protein data bank was selected for comparison. Click Search to get the results of the protein-protein interaction. MicroRNAs targeted relationship by psRNATarget (http://plantgrn.noble.org/psRNATarget/, accessed on 13 November 2022) to predict the default parameters [59]. The interactive network is demonstrated by Cytoscape software. Using RoseTTAFold (https://robetta.bakerlab.org accessed on 13 November 2022) and MIB database (http://bioinfo.cmu.edu.tw/MIB/, accessed on 13 November 2022), the six StSROs protein crystal structures were analyzed [60,61], then PyMOL plugin was used to refine and analyze the predicted protein tertiary structure.

### 4.7. Agrobacterium-Mediated Transient Expression in Potato

The potato transient expression experiment refers to the method of Li et al. in the transformation of *Artemisia annual* L. [62], we make improvements on this basis and operate; the specific operation steps are as follows: (1) The recombinant Agrobacterium pCAMBIA1302-StWRKY6-GFP was spread on LB solid medium containing rifampicin and placed in a 28 °C incubator overnight; (2) after the above LB solid medium was cultured, single colonies were picked and cultured overnight with shaking in LB liquid medium containing rifampicin (28 °C, 2000 rpm); (3) take 10 mL of the bacterial solution and put it in 50 mL of LB liquid medium containing rifampicin and kanamycin, add 20 μM acetosyringone (AS) and 10 mM MES (methyl ester sodium sulfonate), and shake at 28 °C overnight. (4) The bacterial solution was centrifuged for 20 min in a desktop refrigerated centrifuge (4 °C, 5000 rpm), and the precipitate was suspended in a mixture of 100 μM AS, 10 mM MES and 10 mM MgSO_4_. The OD600 value of the bacterial solution was determined when it was between 1.0 and 2.0. For transient expression experiments, we chose potato soil cultured seedlings and potato tissue cultured seedlings, and soaked potato leaves with the above-configured Agrobacterium infection solution (Agrobacterium carries a recombinant plasmid containing the target gene). Using the vacuum pump infection method, after 8–10 min of treatment under the vacuum condition of 0.08 mpa, the hose was quickly extracted to release air, and the operation was repeated for three times. After returning to normal pressure, the whole potato plant was taken out, the surface transformed bacterial liquid was wiped with sterilized filter paper, and then left for 12 h, 24 h, 48 h, and 72 h respectively, and potato leaves at corresponding time points were taken to detect StWRKY6- GFP and StSROs 5/6 expression levels.

### 4.8. In Vitro Culture Test of Potato Leaf and Determination of Related Enzyme Activity

In vitro culture experiment was conducted after transient transfection of potato leaves [63]. First, the leaves were evenly cut into 2 cm pieces and placed on a Petri dish. An equal amount of isolated leaf treatment solution was added to the Petri dish. Hoagland nutrient solution was added with 0 (control), 6, 12, 24, and 100 μmol/L Cd^2+^ solution. After soaking for 12 h, peroxidase (POD) and superoxide dismutase (SOD) activities were detected with relevant kits. The POD activity of potato under Cd stress was detected and calculated using the instruction of solebo (Beijing) Peroxidase Activity Detection Kit (BC0095).

### 4.9. PI staining and Cd Localization Staining

In order to observe the cadmium tolerance of potato leaves before and after transient transfection, we used PI (propidium iodide) staining to observe the cell death [64]. After the potato leaves before and after transient transfection were treated with 100 μmol/L Cd^2+^ solution for 4 days, the leaves were first washed with tap water for 5 min, then washed with ddH_2_O, and finally washed with 0.01 mol/L PBS (pH 7.4). About 0.01 mol/L PBS (phosphate buffered saline) was used to prepare a PI working solution with a final concentration of 50 μg/mL. After the potato leaves were wiped dry, PI staining solution was added for shading and staining for 15 min. The slides were placed on glass slides, covered with a coverslip, and the dead cells were observed using a fluorescence microscope. Cd localization staining: dithizone staining was used (Cd tissue localization staining method) to localize the distribution of Cd in potato roots before and after transient transfection [65]. The instantaneous transfected potato tissue culture seedlings were stressed with 0 μmol/L and 100 μmol/L Cd^2+^ solution for 4 days, the roots of potato tissue culture seedlings were placed in dyeing solution (3 mg dithizone staining, 6 mL acetone, 2 mL deionized water, 10 μL glacial acetic acid), sealed and dyed for 1 h without light. The distribution of Cd in the roots was then observed under a microscope (Yujie, China).

### 4.10. StSRO6 Yeast Functional Complementarity Validation Test

Wild-type BY4741 and Cd-sensitive *Δycf1* were respectively used as positive and negative control to verify whether *Δycf1*-StSRO6 strain could recover its Cd-tolerance ability after heterologous overexpression of potato StSRO6 gene. StSRO6 gene with homologous arm (F: cttggtaccgagctcggatccATGGAGTCAAACT GGGTTGAAGTG; R: tacatgatgcggccctctagaTCAACAATTACTCTCCCCACTCG), the recombinant plasmid *pYES2*-StSRO6 was constructed by one-step homologous recombination with double enzyme digestion (BamHl, XbaI), then the recombinant plasmid was transformed into *Δycf1* strain, accordingly OEStSRO6-*Δycf1* functional complementary type mutant strain was obtained. According to the method of Li et al. (2021), after the three yeast strains obtained were diluted (10^0^, 10^−1^, 10^−2^, 10^−3^, 10^−4^), they were placed in solid and liquid synthetic galactose-uracil (SG-U) deficiency minimal medium containing 0, 15, and 30 μM Cd, respectively, three strains were determined with growth phenotype (7 days after 0, 15, and 30 μM Cd stress), OD values (0, 5, 10, 15, 20, 25, 30, 35, and 45 h), and Cd content (30 μM Cd stress for 7 days).

## Data Availability

Not applicable.

## Supplementary Materials

The following supporting information can be downloaded at: https://www.mdpi.com/article/10.3390/ijms232214318/s1.
