# Supplementary material for: Identification of the Major Effector StSROs in Potato: A Potential StWRKY-SRO6 Regulatory Pathway Enhances Plant Tolerance to Cadmium Stress
Source: Int J Mol Sci. 2022 Nov 18;23(22):14318. doi: 10.3390/ijms232214318 (PMC9698690; doi:10.3390/ijms232214318)
Supplement: Supplementary file 1 [file ijms-23-14318-s001.zip › Supplementary Figures S1-S7.pdf]

Supplementary Materials

Identification of the Major Effector StSRO in Potato: A Potential *StWRKY-SRO6* Regulatory Pathway Enhances Plant Tolerance to Cadmium Stress

Yeqing He [1], Guandi He [1,*], Weijun Tian [2], Fei Lou [1], Zheng Zhou [1], Yao Liu [1], Yule Zhang [1], Tengbing He [1,3,*]

1 College of Agricultural, Guizhou University, Guiyang 550025, China;
2 Institute of Geochemistry, Chinese Academy of Sciences, Guiyang 550081, China
3 Institute of New Rural Development of Guizhou University, Guiyang 550025, China
* Correspondence: gdhe@gzu.edu.cn; tbhe@gzu.edu.cn

Supplementary Figures

Figure S1. Phylogeny, exon length distribution and protein structure of SRO families.

Figure S2. Expression levels of StSROs in different tissues (without Cadmium).

Figure S3. Three-dimensional structure and protein interaction prediction.

Figure S4. *StWRKY6* gene transient transfection experiment.

Figure S5. In vitro culture and determination of cadmium tolerance of potato leaves.

Figure S6. Summary of special characteristics of StSRO5 (VEG) and StSRO6 (MEG).

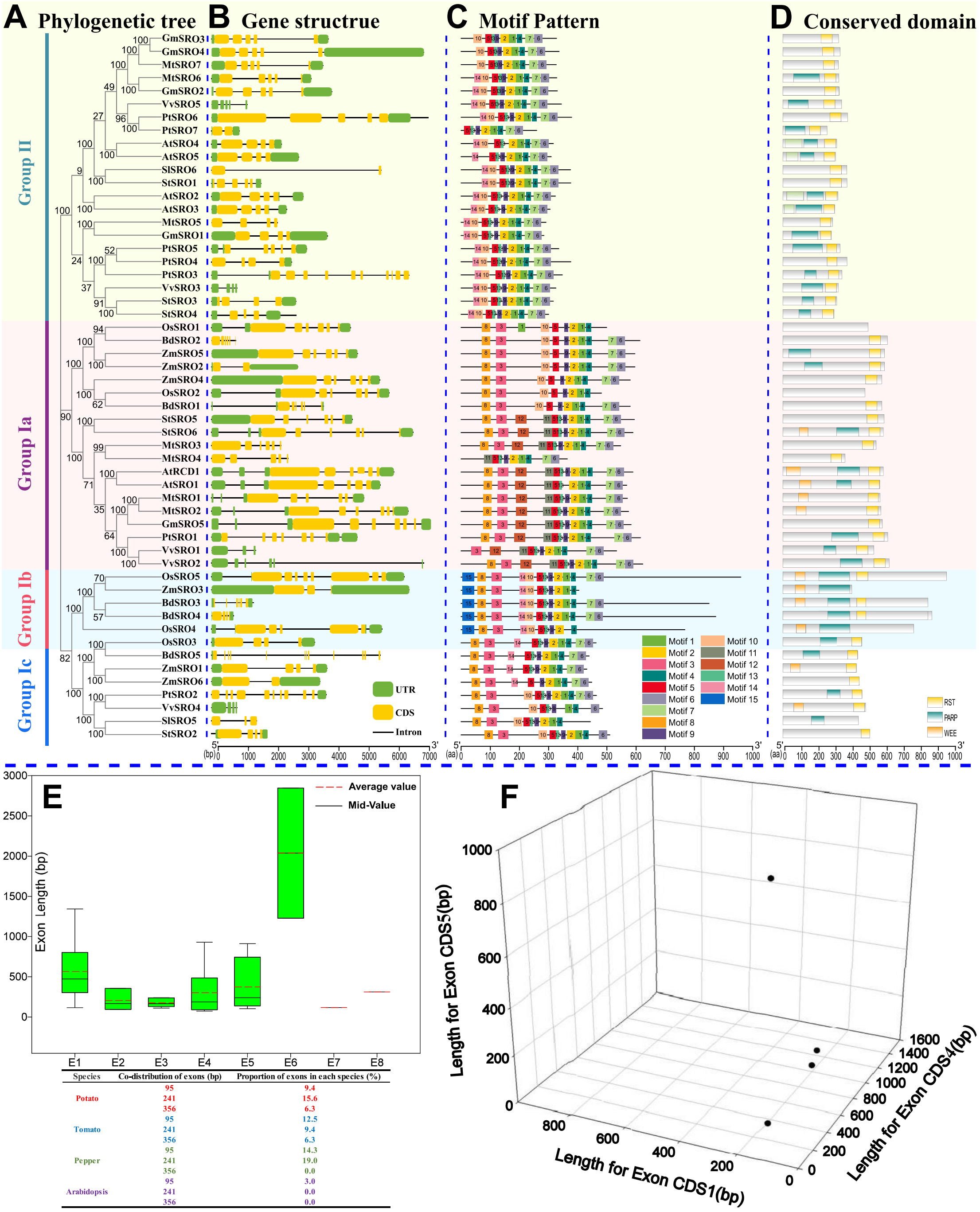

Species	Co-distribution of exons (bp)	Proportion of exons in each species (%)
Potato	95	9.4
	241	15.6
	356	6.3
Tomato	95	12.5
	241	9.4
	356	6.3
Pepper	95	14.3
	241	19.0
	356	0.0
Arabidopsis	95	3.0
	241	0.0
	356	0.0

Figure S1. Phylogeny, exon length distribution and protein structure of SRO families. (**A**) Phylogenetic trees of 73 SROs proteins were constructed using MEGA-X. (**B**) *SRO* genes structures visualized using Tbtools. (**C**) Motif structure of SROs proteins. Untranslated regions (UTR) were represented by green boxes, exons by yellow boxes, and introns by black lines, as detailed in Supplementary Table S7. (**D**) Schematic diagram of SROs functional conservative domain. The RST domain was represented by a yellow box, the PARP domain by a blue box, and the WWE domain by an orange box. (**E**) Analyze the exon lengths using a boxplot in SigmaPlot, where the height of each boxplot represents a range of exon lengths. Grouping 50% of a particular exon value, the mean is shown as a red dotted line and the median as a black solid line. (**F**) Length distributions of the first, fourth and fifth exons of the StSROs, 3D scatter plots plotted using SigmaPlot.olutionary analysis of SRO families in different species.

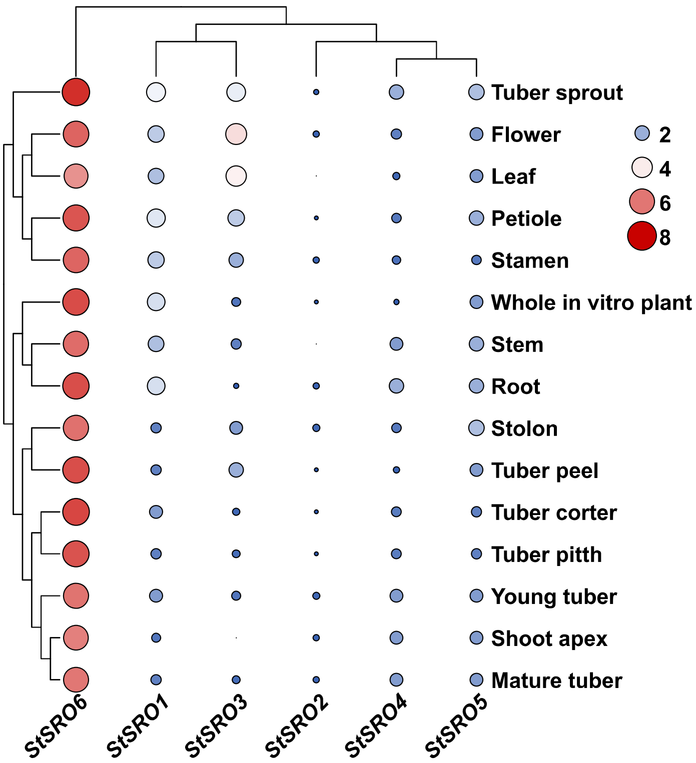

Tissue	*StSRO1*	*StSRO2*	*StSRO3*	*StSRO4*	*StSRO5*	*StSRO6*
Stolon	1.0	0.4	2.0	0.8	4.0	67.0
youngtuber	2.0	0.4	0.7	2.0	2.0	65.0
Flower	5.0	0.3	18.0	1.0	2.0	78.0
Leaf	4.0	0.0	14.0	0.4	2.0	45.0
Shootapex	0.7	0.3	0.0	2.0	2.0	56.0
Petiole	8.0	0.1	5.0	0.8	3.0	94.0
Stem	4.0	0.0	1.0	2.0	3.0	71.0
Mature tuber	1.0	0.3	0.5	2.0	2.0	62.0
Root	7.0	0.3	0.2	3.0	3.0	102.0
Stamen	5.0	0.3	3.0	0.6	0.8	77.0
Tuber peel	1.0	0.1	3.0	0.3	2.0	102.0
Tuber corter	2.0	0.1	0.4	0.9	1.0	112.0
Tuber pitth	1.0	0.1	0.5	0.9	1.0	96.0
Tubers prout	10.0	0.2	9.0	3.0	4.0	148.0
Whole in vitro plant	7.0	0.1	0.7	0.2	2.0	104.0

TPM values of *StSRO*s in different potato tissues

Figure S2. Expression levels of StSROs in different tissues (without Cadmium). The data were downloaded from the gene expression profile database (Array Express, https://www.ebi.ac.uk/arrayexpress). The heat map was generated using Tbtools software. The gradient color from red to blue is the transcription level of each *StSRO*s, with red and gray-blue representing high and low expression levels, respectively. The circle size also represents the corresponding level of expression. The table on the right of the figure shows TPM values of *StSRO*s in different potato parts.

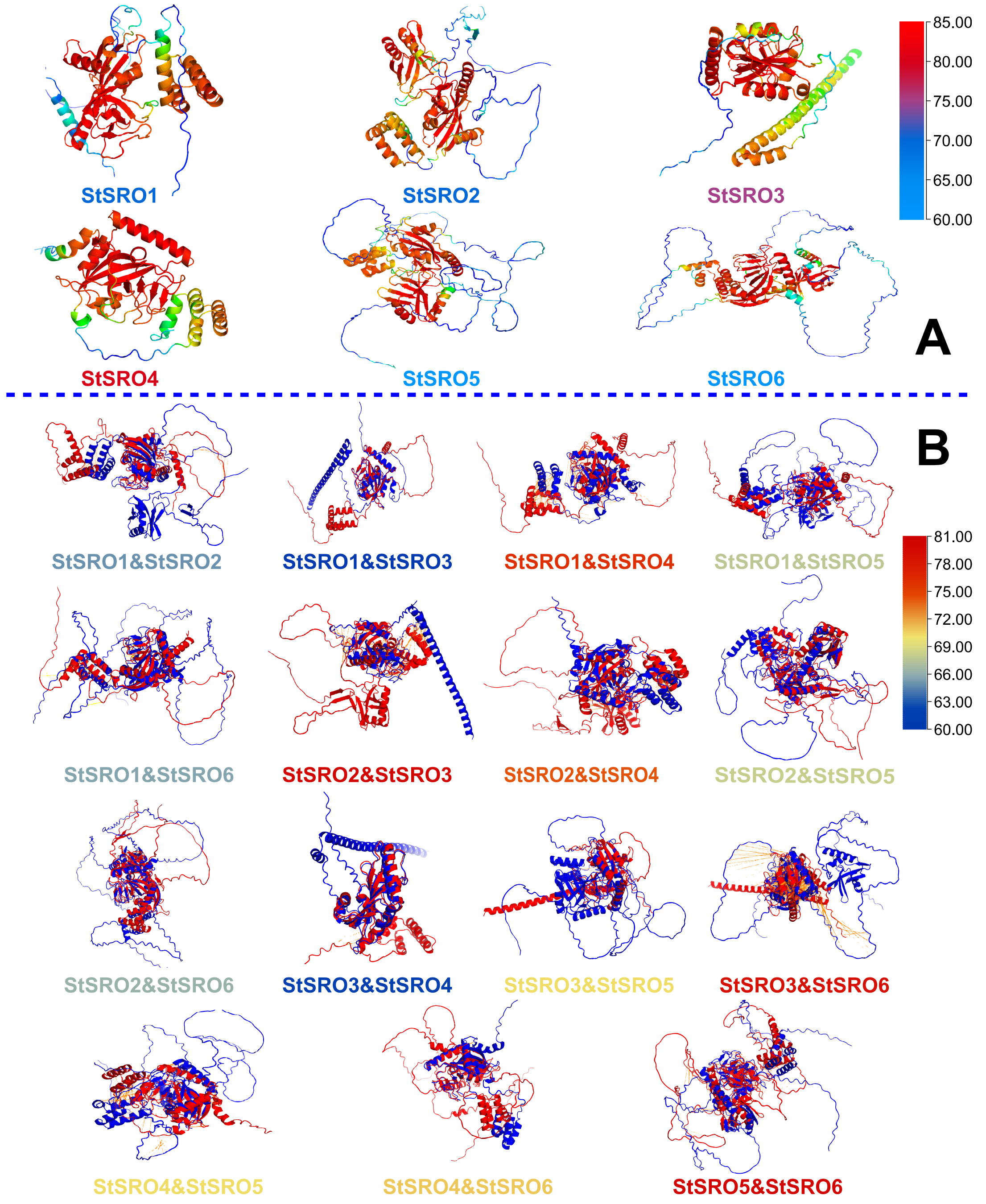

Figure S3. Three-dimensional structure and protein interaction prediction. (**A**) Three-dimensional structure prediction of StSROs proteins. (**B**) StSROs proteins interaction prediction, the closer the color is to red, the higher the accuracy of structure prediction.

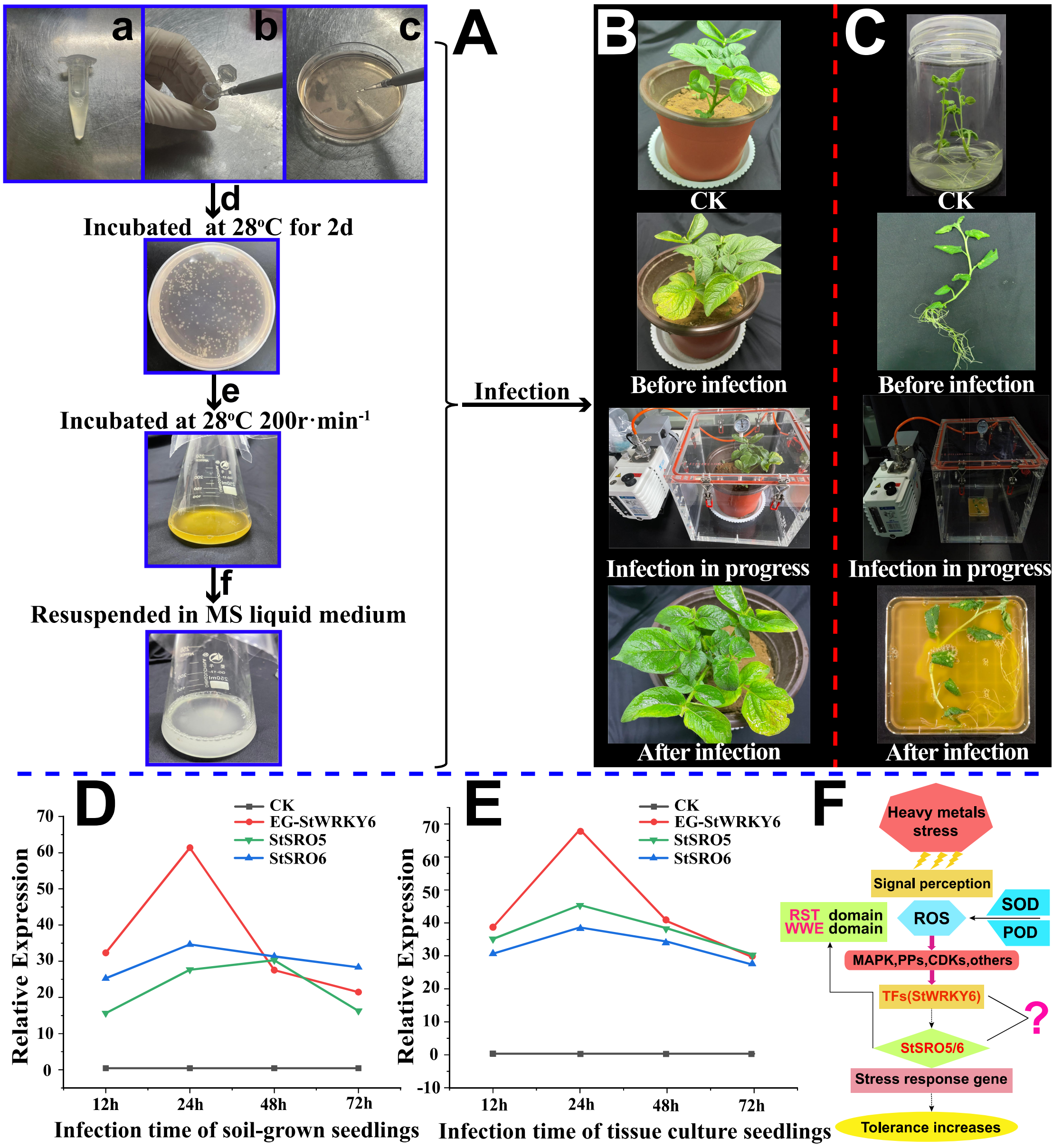

Figure S4. *StWRKY6* gene transient transfection experiment. (**A**) Preparation of strains of agrobacterium infection. a: Bacteria are stored in glycerol at -80 °C; b: Inoculate ring soaked bacteria: c: Bacterial fluid was underline in LB solid medium containing antibiotics; d: Incubate for two days in a constant temperature incubator at 28 °C; e: The agrobacterium strains oscillated in LB liquid culture containing rifampicin until OD600 reached 0.6; f: The bacterial solution was centrifuged and placed in MS liquid medium to resuspend the strain. (**B**) Plant phenotypes of potato seedlings cultured in soil before, during and after transient transfection. (**C**) Plant phenotypes of potato tissue culture seedlings before, during and after transient transfection. (**D**) The expression levels of *StWRKY6* and *StSROs 5/6* genes in the leaves of potato soil-grown seedlings at different times were determined, as detailed in Supplementary Table S13a. (**E**) The expression levels of *StWRKY6* and *StSROs 5/6* genes in the leaves of potato tissue culture seedlings at different times were determined, as detailed in Supplementary Table S13b. (**F**) It is speculated that there may be a strip of potential StWRKY6-SROs 5/6 regulatory pathway.

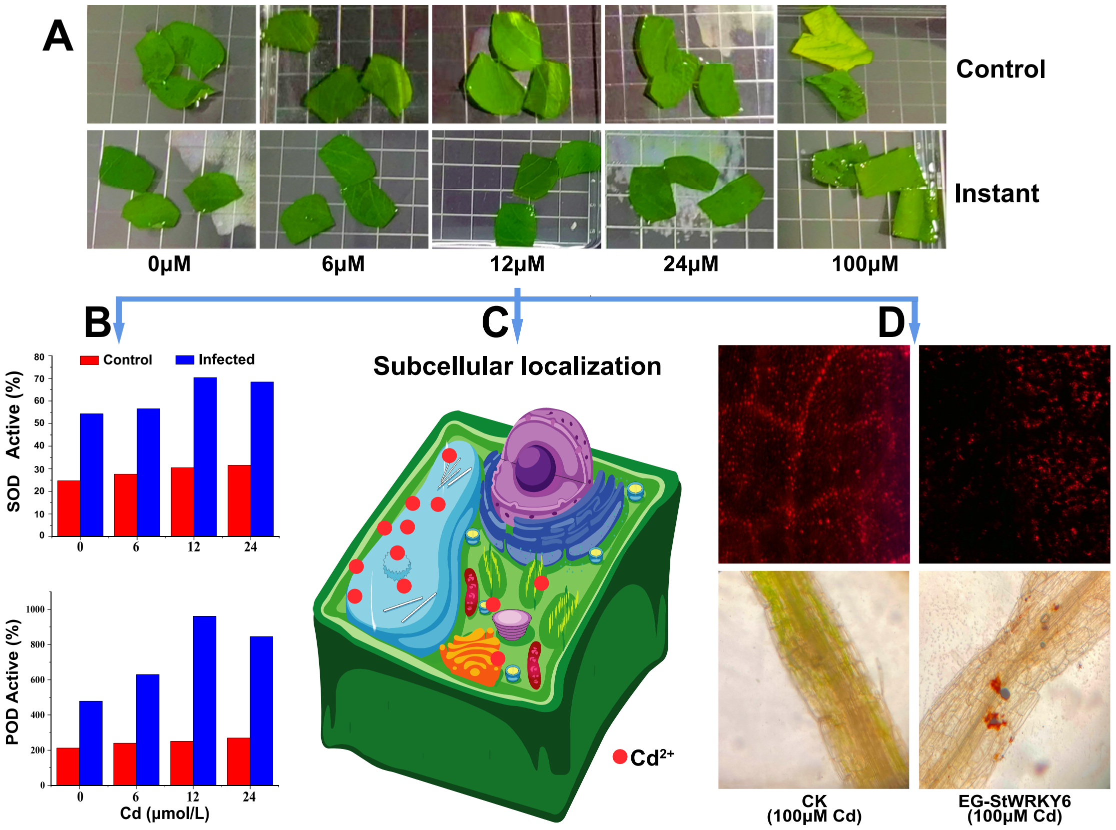

Figure S5. In vitro culture and determination of cadmium tolerance of potato leaves. (**A**) In vitro culture of potato leaves under different Cd^{2+} stress, Control represents leaf growth under normal conditions (without cadmium stress), Instant represents Cd stress at different concentrations. (**B**) Superoxide dismutase (SOD) and peroxidase (POD) activity detection. (**C**) Schematic diagram of Cd^{2+} destruction of plant cells. (**D**) PI staining of potato leaves and dithizone staining of root system of potato (tissue culture).

Function	StSRO5 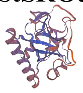	StSRO6 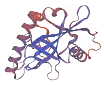
**Subcellular localization**	Nucleus	
**Architectural feature**	RST	WWE-PARP-RST
**Co-expression**	*StSRO5* & *StSRO6* (Cd)	
**Positive response**	Cd	
	Zn	Ni
**Interactions between proteins**	*StSRO5* & *StSRO6*	

Figure S6. Summary of special characteristics of StSRO5 (VEG) and StSRO6 (MEG).

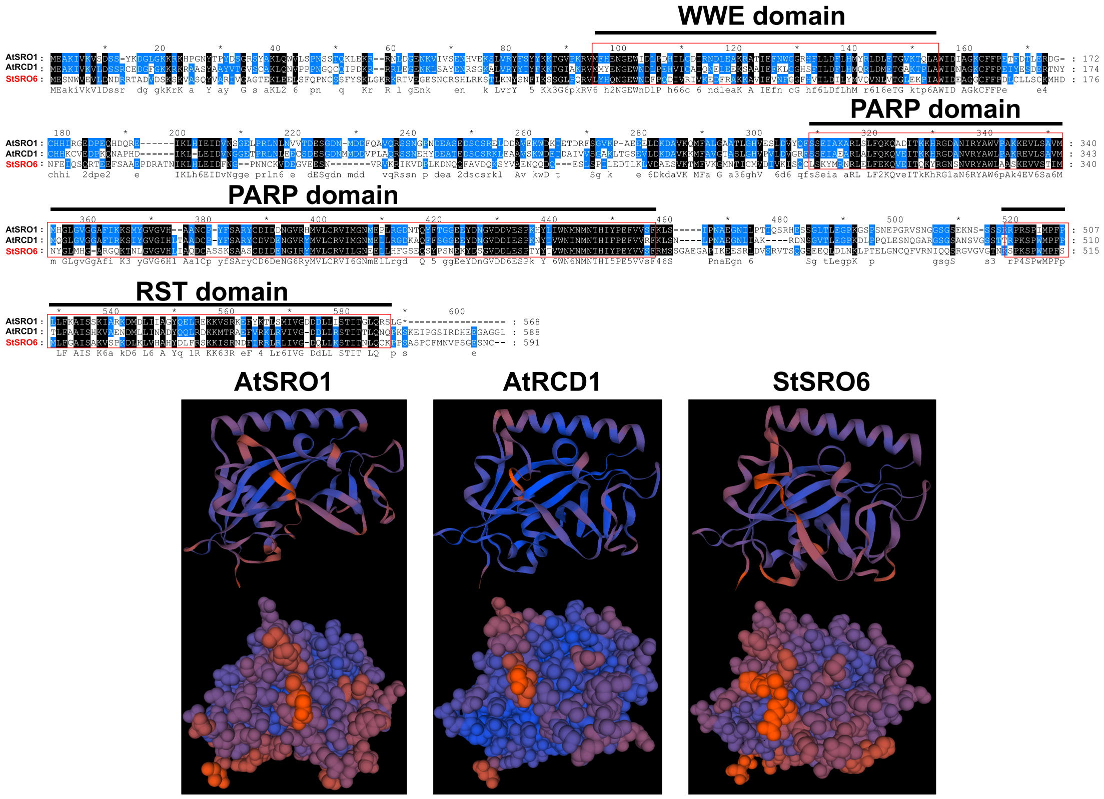

Figure S7. Sequence analysis of the *Solanum tuberosum* SRO family. Blue and Black colors indicate similar amino acid sequences; Potato and Arabidopsis proteins were imported into clustalx-2.0.11 for comparison. Conserved domain predictions were made using Smart (http://smart.embl-heidelberg.de/). The results were further analysis by using GeneDoc software.Blue indicates similarity > 80% and gray indicates similarity between 60% and 80%.
